# Sirtuin 1 overexpression in mice preserves insulin and thermogenic responses in subcutaneous inguinal white adipose tissue under proinflammatory conditions

**DOI:** 10.1007/s13105-025-01109-3

**Published:** 2025-08-04

**Authors:** Patricia Vázquez, Carmen Escalona-Garrido, Nuria Pescador, Ana B. Hitos, Daniel González-Moreno, Ángela de Benito-Bueno, Elena Sierra-Filardi, Patricia Boya, Ana Montero-Pedrazuela, Ana Guadaño-Ferraz, Ángela M. Valverde

**Affiliations:** 1 Instituto de Investigaciones Biomédicas “Sols-Morreale” (IIBm, CSIC- UAM), Madrid, Spain; 2 CIBER de Diabetes y Enfermedades Metabólicas (CIBERDEM), ISCIII, Madrid, Spain; 3 Departamento de Bioquímica y Biología Molecular, Facultad de Medicina, Universidad Complutense de Madrid, Madrid, Spain; 4 Departamento de Biología Celular y Molecular, Centro de Investigaciones Biológicas Margarita Salas (CIB, CSIC), Madrid, Spain

**Keywords:** iWAT, SIRT1, Browning, UCP-1, Inflammation, Insulin signaling, Obesity

## Abstract

Activation of brown adipose tissue (BAT) or subcutaneous adipose tissue (iWAT in mice) is a strategy to regulate metabolic homeostasis. The NAD^+^-dependent deacetylase Sirtuin 1 (SIRT1) plays an essential role in energy metabolism and inflammation and is a promising target to tackle obesity and associated comorbidities. We have previously reported the beneficial effect of moderate SIRT1 overexpression in protecting mice against inflammation-induced insulin resistance and impaired BAT thermogenesis. Here, we investigated the effect of an inflammatory environment on insulin sensitivity and thermogenic capacity in iWAT from wild-type (WT) or SIRT1 overexpressing mice (Sirt1^*Tg+*^). We also analyzed in vitro responses to insulin and norepinephrine (NE) in subcutaneous white adipocytes (iWA) from both genotypes under proinflammatory conditions. Results showed higher UCP-1 levels in iWAT from Sirt1^*Tg+*^ mice under thermoneutral conditions compared to WT mice, an effect also found in vitro in differentiated iWA. Cold-induced UCP-1 expression and insulin-induced Akt phosphorylation levels were reduced in iWAT from WT mice upon in vivo bacterial lipopolysaccharide (LPS) injection. However, these reductions were attenuated in iWAT from Sirt1^*Tg+*^ mice. Likewise, in iWA exposed to the conditioned medium from LPS-stimulated Raw 264.7 macrophages (CM-LPS) both insulin signaling and NE-induced UCP-1 expression levels were preserved only in cells overexpressing SIRT1. LPS or CM-LPS increased SIRT1 levels in iWAT or iWA, respectively, an effect more evident upon SIRT1 overexpression. Collectively, our results suggest a SIRT1-dependent anti-inflammatory compensatory response that likely protects iWAT from the deleterious effects of inflammation.

**Supplementary Information:**

The online version contains supplementary material available at 10.1007/s13105-025-01109-3.

## Key points

iWAT and iWA of Sirt1^*Tg+*^ mice show higher UCP-1 levels under basal conditions.

iWAT and iWA from Sirt1^*Tg+*^ mice preserve insulin and thermogenic responses in a proinflammatory environment.

SIRT1 is further increased in iWAT and iWA from Sirt1^*Tg+*^ mice under proinflammatory conditions.

**Supplementary Information:**

The online version contains supplementary material available at 10.1007/s13105-025-01109-3.

## Introduction

Chronic low-grade inflammation leads to metabolic disorders such as obesity or type 2 diabetes [1]. In obese patients, changes in the gut microbiota increase intestinal permeability leading to an elevation of circulating levels of bacterial lipopolysaccharide (LPS) which impairs the function of relevant metabolic tissues involved in the control of insulin action and energy balance [2–4]. Furthermore, this low-grade chronic inflammation characterized by immune cell infiltration in tissues contributes to the expansion of white adipose tissue (WAT) which is a key feature of obesity [5]. Under these conditions, the proinflammatory cytokines derived from resident and recruited macrophages, among other immune cells, have a deleterious effect on insulin signaling resulting in excessive WAT lipolysis and reduced glucose uptake, thereby contributing to the insulin resistance observed in obese patients [1, 5]. On the other hand, emerging evidence suggests that epididymal and subcutaneous WAT (eWAT and inguinal or iWAT in mice) play distinct roles in the regulation of energy homeostasis, with eWAT being mainly responsible for energy storage whereas iWAT, the so-called brite or beige adipose tissue, has the capacity to contribute to energy expenditure [4, 6]. In addition, iWAT is characterized by improved insulin sensitivity compared to eWAT [7, 8]. Of relevance, iWAT has been proposed to be the closest mouse equivalent of human brown adipose tissue (BAT) [9]. Under conditions of obesity-associated low-grade inflammation, infiltration of immune cells creates a deleterious inflammatory microenvironment that alters the brown-white balance of iWAT and impairs the extent of browning in these depots [10, 11]. In this regard, Sanchez-Infantes et al. reported an impairment of iWAT browning in mice treated with oncostatin, a cytokine that is elevated in obesity [12]. Thus, understanding how low-grade inflammation may affect iWAT plasticity deserves more in depth investigation.

Sirtuins (SIRT1-7) are nicotinamide adenine dinucleotide (NAD^+^) class III histone deacetylases involved in the control of several cellular physiological processes, including cell survival, senescence, aging and metabolic homeostasis [13, 14]. Several studies have investigated the role of the SIRT1 family member in adipose tissue as this organ appears to be an important target of this enzyme [15, 16]. In a previous work we demonstrated the benefit of moderate SIRT1 overexpression in mice in improving glucose homeostasis by enhancing BAT function [17]. Regarding iWAT, SIRT1 has been shown to deacetylate the transcription factor peroxisome proliferator-activated receptor γ (PPARγ), resulting in the induction of adipose-related genes [18]. In the context of obesity-related inflammation, the work of Hui et al. showed that mice with adipocyte-selective deletion of SIRT1 are more susceptible to diet-induced insulin resistance, an effect associated with increased numbers of adipose tissue-resident macrophages of the pro-inflammatory M1 subtype [19]. Mechanistically, this study showed that, in adipocytes, SIRT1 deacetylates the transcription factor NFATc1 (nuclear factor of activated T cells 1) and enhances its binding to the interleukin 4 (IL-4) promoter, thereby increasing IL-4 expression. In another study, SIRT1 knockdown in WAT resulted in nuclear localization of nuclear factor-kappa B (NF-κB), an effect associated with a global reduction in H3K9 deacetylation [20]. Furthermore, overexpression of SIRT1 prevents the accumulation of adipose tissue macrophages in mice fed a high fat diet (HFD) [20].

We have recently reported the beneficial effect of moderate SIRT1 overexpression in protecting mice against LPS-induced pro-inflammatory signaling, insulin resistance, and reduction of thermogenic responses in BAT [21], suggesting the potential benefit of SIRT1 activators in combating metainflammation in this tissue. The present study aimed to investigate the role of SIRT1 in the effect of a pro-inflammatory environment on the insulin sensitivity and thermogenic capacity of iWAT. Furthermore, we elucidated the role of SIRT1 in the cell-autonomous effects of subcutaneous inguinal white adipocytes (iWAT) under pro-inflammatory conditions.

## Materials and methods

### Animal experimental protocol

C57Bl/6J male mice overexpressing SIRT1 (Sirt1^*Tg+*^) and their respective wild-type controls (WT), provided by Dr. Manuel Serrano (CNIO, Madrid, Spain), were housed at the animal facilities of Instituto de Investigaciones Biomédicas Sols-Morreale (IIBm, CSIC/UAM, Madrid) under controlled conditions of 12 h:12 h light-dark cycles at 22ºC and 45–55% humidity and allowed free access to a standard rodent chow diet (A04, U8220G10R, SAFE) and water. Animal procedures were approved by the Ethics Committee of the Spanish National Research Council (CSIC) and Comunidad de Madrid (Spain) (reference PROEX007/19 approved on 02/11/2019).

To study the thermogenic responses in iWAT under basal conditions, 3–5 month old wild-type (WT) and Sirt1^*Tg+*^ mice were housed at 28ºC for one week. To analyze the effect of cold exposure, mice were housed at 28ºC for one week and then exposed to the cold (4ºC) for 6 h. To analyze the effect of LPS, WT and Sirt1^*Tg+*^ mice were housed at 28ºC for one week before receiving a single intraperitoneal (i.p.) injection of LPS (2 mg/kg, tlrl-eblps, InvivoGen) or saline 24 h before sacrifice. A subset of mice was exposed to 4ºC for the final 6 h.

To analyze insulin signaling in vivo, WT and Sirt1^*Tg+*^ mice were fasted for 4 h, injected i.p. with 0.75 U/kg human recombinant insulin (Actrapid, Novo Nordisk), and sacrificed 20 min later. To analyze insulin responses under pro-inflammatory conditions, WT and Sirt1^*Tg+*^ mice were injected with 2 mg/kg LPS 24 h before sacrifice and fasted for the last 4 h. The control group received saline. After 24 h, mice were injected with 0.75 U/kg insulin (i.p.) and sacrificed after 20 min. A subset of mice did not receive insulin injections. Mice were sacrificed and iWAT depots were stored at -80 °C until use. The number of animals or samples used are indicated in the figure legends.

### Histological analysis and immunostaining

iWAT was fixed in 4%-paraformaldehyde (PFA,16005, Merck Life Science S.L.U.) for 24 h. Tissue was dehydrated with ascending ethanol solutions, incubated with xylene and then embedded in paraffin. Tissue sections of 5 μm were deparaffinized and hydrated and the slides were stained with Mayer’s hematoxylin solution and eosin (H&E) (MHS32-1 L, 1.15935.0025, Merck, respectively) for 1 min.

For UCP-1 immunohistochemistry (IHC), iWAT sections were deparaffinized and after heating for 20 min in antigen retrieval solution (100 mM sodium citrate buffer (pH 6.0) and 0.05% Tween-20), sections were blocked with 6% BSA and 2% horse serum (NHS) in PBS-0.1% Triton X-100 for 1 h at room temperature (RT). The sections were then incubated with primary anti-UCP-1 antibody (Ab10983, Abcam) (diluted 1:500) overnight at 4ºC. After washing, sections were incubated with biotinylated secondary anti-rabbit antibody (1:250) (BA-1100, Vector Laboratories) for 1 h at RT. Sections were stained with DAB peroxidase substrate kit (SK-4100, Vector Laboratories) and counterstained with hematoxylin (H3136, Merck). The microphotographs were captured with an Axiophot light microscope (Zeiss) using a 40X objective.

### T_3_ radioimmunoassay in iWAT

Extraction of triiodothyronine (T_3_) from iWAT and T_3_ determinations were conducted as previously described by Escalona et al. for T_3_ detection in BAT extracts [21] based on the previous work of Morreale de Escobar et al. [22]. High specific activity ^125^I-T_3_ (3000 µCi/µg) was labeled with ^125^I (NEZ033A; Revvity) using 3,5-Diiodo-L-thyronine (D0629; Merck) as substrate, as previously described by Obregon* et al.* [23] with minor modifications [24].

### Generation of pro-inflammatory conditioned medium (CM) from macrophages

Raw 264.7 murine macrophages were maintained in Roswell Park Memorial Institute (RPMI) medium supplemented with 100 U/mL penicillin, 100 µg/mL streptomycin, 10 mM HEPES, 2 mM L-glutamine, and 10% fetal bovine serum (FBS). Macrophages were grown until 95–100% confluence was reached. Then the medium was changed to RPMI-2% FBS for 1 h and cells were treated with 100 ng/mL LPS for 5 h or left untreated. The culture medium was then removed and the cells were washed with phosphate-buffered saline (PBS) and cultured in RPMI-2% FBS medium for 17 h to obtain LPS or control conditioned medium (CM-LPS or CM-CTR, respectively). The CM were filtered and supplemented with FBS (final 5%) before the addition to iWA cultures.

### Generation and differentiation of immortalized inguinal white preadipocyte cell lines from WT and Sirt1^Tg+^ mice

WT and Sirt1^*Tg+*^ inguinal preadipocytes (iWA-WT and iWA-Sirt1^*Tg+*^, respectively) were isolated from iWAT from WT and Sirt1^*Tg+*^ mice and immortalized using the same protocol previously described to generate immortalized brown adipocyte cell lines [17, 21]. Immortalized iWA preadipocytes were maintained in DMEM/F-12 (D19800, Thermofisher) supplemented with 100 U/mL penicillin, 100 mg/mL streptomycin, 10 mM HEPES, and 10% FBS. Two different protocols were tested for adipocyte differentiation.

Protocol 1: the cells were first grown in growing medium 1 (DMEM/F-12 supplemented with 10% FBS, 1 nM T_3_ (T6397, Merck), and 850 nM insulin (I0516, Merck), until confluence was reached. Then, cells were stimulated for 36 h with growing medium 1 supplemented with 5 µM dexamethasone (D1756, Merck), 1 µM rosiglitazone (R2408, Merck), 0.5 mM isobutylmethylxanthine (IBMX) (I5879, Merck), and 125 nM indomethacin (I7378, Merck) (differentiation medium 1), as previously described by Armani et al. [25]. The cells were then cultured back into growing medium 1 supplemented with 1 µM of rosiglitazone for 3 additional days.

Protocol 2 (adapted from [26]): cells were plated in growing medium 2 (DMEM/F-12 supplemented with 10% FBS, 3 µM T_3_ and 850 nM insulin) until confluence was reached. Then, cells were stimulated for 36 h with growing medium 2 supplemented with 35 nM dexamethasone and 10 µM rosiglitazone (differentiation medium 2). The cells were then maintained in DMEM/F-12 supplemented with 10% FBS, 10 µM rosiglitazone, 3 µM T_3_, and 850 nM insulin for 3 additional days.

The key differences between the two media are the concentrations of T_3_ (1 nM in medium 1 *versus* 3 µM in medium 2) and rosiglitazone (1 µM in medium 1 *versus* 10 µM in medium 2).

### Insulin and norepinephrine responses in differentiated iWA

For insulin signaling studies, WT and Sirt1^*Tg+*^ iWA differentiated with medium 2 were serum starved in DMEM/F-12 for 1 h and then stimulated with 100 nM insulin for 15 min. To conduct experiments in iWA under proinflammatory conditions, cells were pre-incubated with CM-CTR or CM-LPS collected from Raw 264.7 macrophages during 18 h. Then cells were serum starved for 1 h prior to stimulation with 100 nM insulin for 15 min.

For the analysis of thermogenic capacity, differentiated iWA (medium 2) were incubated with DMEM/F-12 supplemented with 5% FBS for 1 h, and then stimulated with 5 µM norepinephrine (NE) (A9512, Merck) for 18 h. To analyze this response under proinflammatory conditions, iWA were incubated with CM-CTR or CM-LPS with or without 5 µM NE during 18 h.

After the treatments, the culture medium was removed and the cells were lysed and total protein extracts were analyzed by Western blot.

### Oil red-O staining

iWA were fixed with 4%-PFA during 10 min and then washed twice with PBS and incubated with oil Red O (O0625, Merck) working solution (3 mg/ml in 30:20 isopropanol: water mixed solution) during 1 h at RT in shaking. After removal of the oil red O solution from the wells, cells were washed 3 times with 60% isopropanol and allowed to dry. Microphotographs were acquired with a bright field microscope (Eclipse TS100, Nikon and Canon Nikon ELWD 0.3/OD75) with 10X or 40X objectives.

### Protein extraction and western blotting

Proteins from WT and Sirt1^*Tg+*^ iWAT were extracted with lysis buffer (50 mM Hepes pH 7.5, 1% Triton X-100 (T8787, Merck), 50 mM tetrabasic sodium pyrophosphate (P8010, Merck), 0.1 M sodium fluoride (NaF, S7920, Merck), 10 mM EDTA, 10 mM o-sodium vanadate (S6508, Merck), 1 mM phenylmethanesulfonyl fluoride (PMSF, P7626, Merck), and 10 µg/mL protease inhibitor cocktail (P-8340, Merck)) at pH 7.4–7.6, using a tissue homogenizer (0003737000, IKA). After centrifugation at 98,560 x g for 40 min at 4 °C, the supernatant was centrifuged at 16,000 x g for 1 h at 4ºC and the resulting supernatant was collected and frozen.

For obtaining protein extracts from iWA, cells were lysed with lysis buffer (10 mM o Tris pH 7.5, 5 mM EDTA, 50 mM HCl, 30 µM sodium pyrophosphate, 50 mM NaF, 100 mM o-vanadate sodium, 1% Triton X-100, 1 mM PMSF, and 10 µg/mL protease inhibitors) at pH 7.4–7.6. The cells were then centrifuged at 11,200 × *g* for 7 min at 4 °C and the supernatant was collected and frozen.

Protein levels were quantified using a BCA Protein Assay Kit (23225, Thermo Fisher Scientific). Protein extracts were submitted to 8–15% SDS polyacrylamide gel electrophoresis (SDS-PAGE) and blotted (PVDF membranes, IPVH00010; Merck). After blocking with blocking solution (5% BSA in Tris-buffered saline pH 7.5 containing 0.05% Tween-20 (TBS-T)) for 1 h at RT, the membranes were incubated o/n with primary antibodies at 4ºC. Antibodies used were fatty acid synthase (FAS) (610962, 1:1000, BD Biosciences), phospho-AKT (ser 473) (9271, 1:2000, Cell Signaling, NEB, UK), AKT (sc-8312, 1:2000, Santa Cruz Biotechnology), SIRT1 (07-131, 1:2000, Merck), UCP-1 (ab10983, 1:1000 Abcam), Vinculin (sc-73614, 1:10000, Santa Cruz), Tubulin (T5168, 1:10000 Merck), and GAPDH (sc-365062, 1:5000; Santa Cruz). Then blots were incubated with the appropriate secondary antibody for 1 h a RT (goat anti-mouse, sc-2005, Santa Cruz, and goat anti-rabbit, A120-108P, Bethyl). Signal intensity was normalized using vinculin, tubulin or GAPDH as loading controls. The membranes were developed with a chemiluminescent substrate (Clarity Western ECL Substrate, 170–5060, Bio-Rad) in a ChemiDOC imager (Bio Rad laboratories) or radiographic films. The densitometry values were determined using ImageJ software (NIH, USA).

### Quantitative real-time PCR analysis

Total RNA from iWAT was extracted using TRIzol™ reagent (15596026, Thermo Fisher Scientific). Reverse transcription was performed using a High-Capacity cDNA Reverse Transcription Kit using random primers and Superscript III enzyme 18,080,093 according to the manufacturer’s instructions (4368813, Thermo Fisher Scientific). Quantitative real-time PCR (qRT-PCR) was performed in a 7900 HT-Fast real-time PCR (Life Technologies) with TaqMan Universal PCR Master Mix. qRT-PCR was carried out at the Genomics Service at IIBm using 5 ng of cDNA. The *Ucp1*,* Sirt1* and *Tbp* probes were supply by Applied Biosystems (Mm01244861_m1, Mm00490758_m1 and Mm00446973_m1 respectively). The relative changes in gene expression were calculated using the 2^−ΔΔCt^ (cycle threshold) quantification method normalized to the expression levels of the *Tbp* (TATA-binding protein) gene. In the in vivo studies the control group used to calculate the relative changes was iWAT-WT at thermoneutrality (28ºC). In cultured iWA, *Sirt1* mRNA expression in iWA-Sirt1^*Tg+*^ was referred to that of iWA-WT.

### Statistical analysis

GraphPad Prism software versions 5.0 and 8.0 (GraphPad Software, San Diego, CA, USA) were used for the statistical analyses. Comparisons between groups were performed with two-way analysis of variance (Two-ANOVA) or with non-parametric Mann-Whitney test. All graphs were generated using GraphPad Prism. Data are represented as mean ± standard error of the mean from at least three independent experiments. The number of animals or samples used are indicated in the figure legends. A *p*-value < 0.05 was considered statistically significant (*^,#^*p* < 0.05; **^,##^*p* < 0.01; ***^,###^*p* < 0.001; and ****^,####^*p* < 0.0001).

## Results

### Effect of Sirt1 overexpression in UCP-1 levels in iWAT from mice housed at thermoneutrality or after cold exposure

**Fig. 1:**
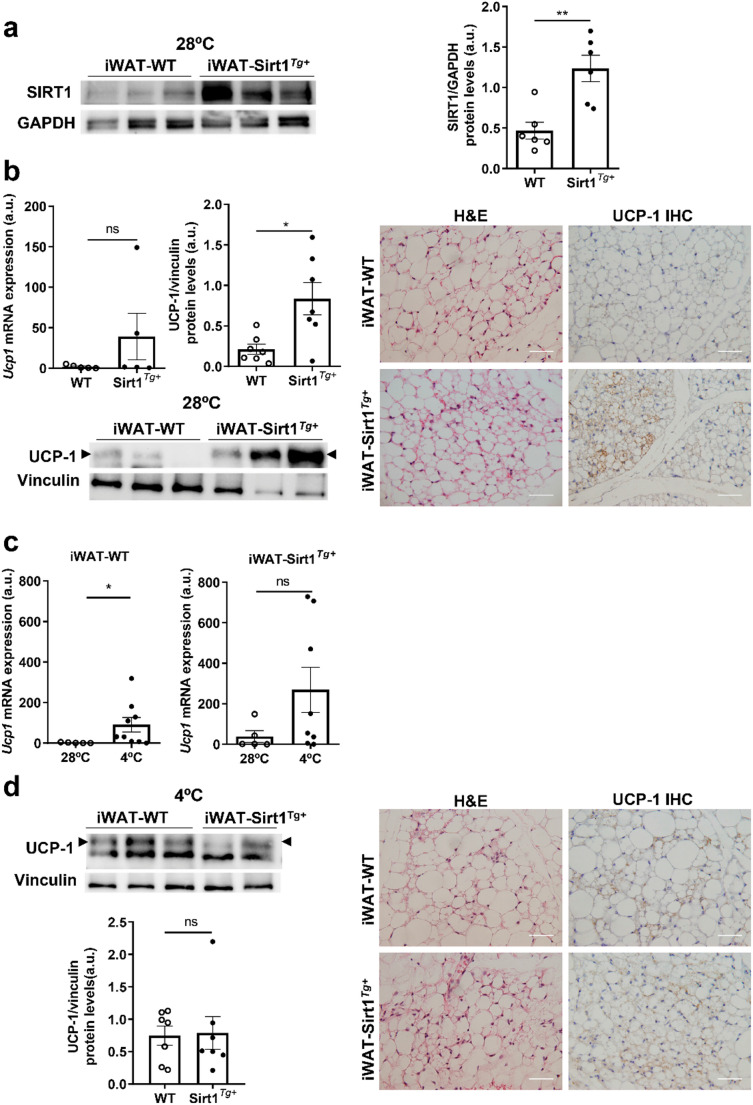
iWAT from Sirt1^*Tg+*^ mice showed higher UCP-1 levels at thermoneutrality. a) Representative SIRT1 Western blot in mice housed at thermoneutrality (28ºC) and quantification (n=6 mice/group). b) *Ucp1* mRNA expression (n=5 mice/group), UCP-1 protein levels and representative UCP-1 Western blot in iWAT from WT and Sirt1^*Tg+*^ at thermoneturality (28ºC) (n = 7 mice/group). The right panel shows representative H&E staining and representative UCP-1-immunohistochemistry images in iWAT from mice housed at 28ºC. c) *Ucp1* mRNA expression in iWAT from WT and Sirt1^*Tg+*^ mice housed at thermoneutrality exposed or not to the cold (4ºC) for 6 h (n= 5-9 mice/group). d) Representative UCP-1 Western blot and quantification in iWAT from mice expose to 4ºC for 6 h (n=7 mice/group). Arrowheads indicate the quantified band. The right panel shows representative H&E staining and UCP-1-immunohistochemistry images of iWAT after cold exposure conditions. Scale bars 50 μm. Vinculin or GAPDH were used as loading controls in Western blots. Data are expressed as mean±SEM. Statistical analyses were performed with Mann–Whitney U test. *p<0.05, **p<0.01.

We first confirmed SIRT1 overexpression in iWAT from Sirt1^*Tg+*^ mice housed at 28ºC for one week by Western blot (Fig. 1a). In order to assess the relevance of SIRT1 overexpression in this fat depot, we first analyzed UCP-1 levels in WT and Sirt1^*Tg+*^ mice housed at thermoneutrality. Interestingly, iWAT from Sirt1^*Tg+*^ mice presented areas with smaller adipocytes (Fig. 1b). Moreover, *Ucp1* mRNA levels showed a trend to an increase in iWAT from Sirt1^*Tg+*^ mice compared to WT mice, an effect that was more evident in UCP-1 protein levels as shown by Western blot and IHC (Fig. 1b).

Since previous studies have demonstrated the contribution of iWAT in maintaining body temperature upon cold exposure [27–29], we analyzed the impact of SIRT1 overexpression in modulating UCP-1 levels under this condition. To achieve this, WT and Sirt1^*Tg+*^ mice were housed at 28ºC during one week and then exposed to cold (4ºC) for 6 h. As expected, *Ucp1* mRNA levels markedly increased in iWAT from WT mice upon cold exposure (Fig. 1c). In iWAT from Sirt1^*Tg+*^ mice, *Ucp1* mRNA expression levels were higher than those of WT mice under thermoneutral conditions, but did not increase significantly upon cold exposure (Fig. 1c). In this regard, similar UCP-1 protein levels were observed in iWAT from both genotypes of mice exposed to 4ºC (Fig. 1d). As occurred in thermoneutral conditions, smaller adipocytes were observed in iWAT from Sirt1^*Tg+*^ mice expose to the cold.

### SIRT1 overexpression protects against the drop of UCP-1 levels in iWAT in mice injected with LPS

**Fig. 2:**
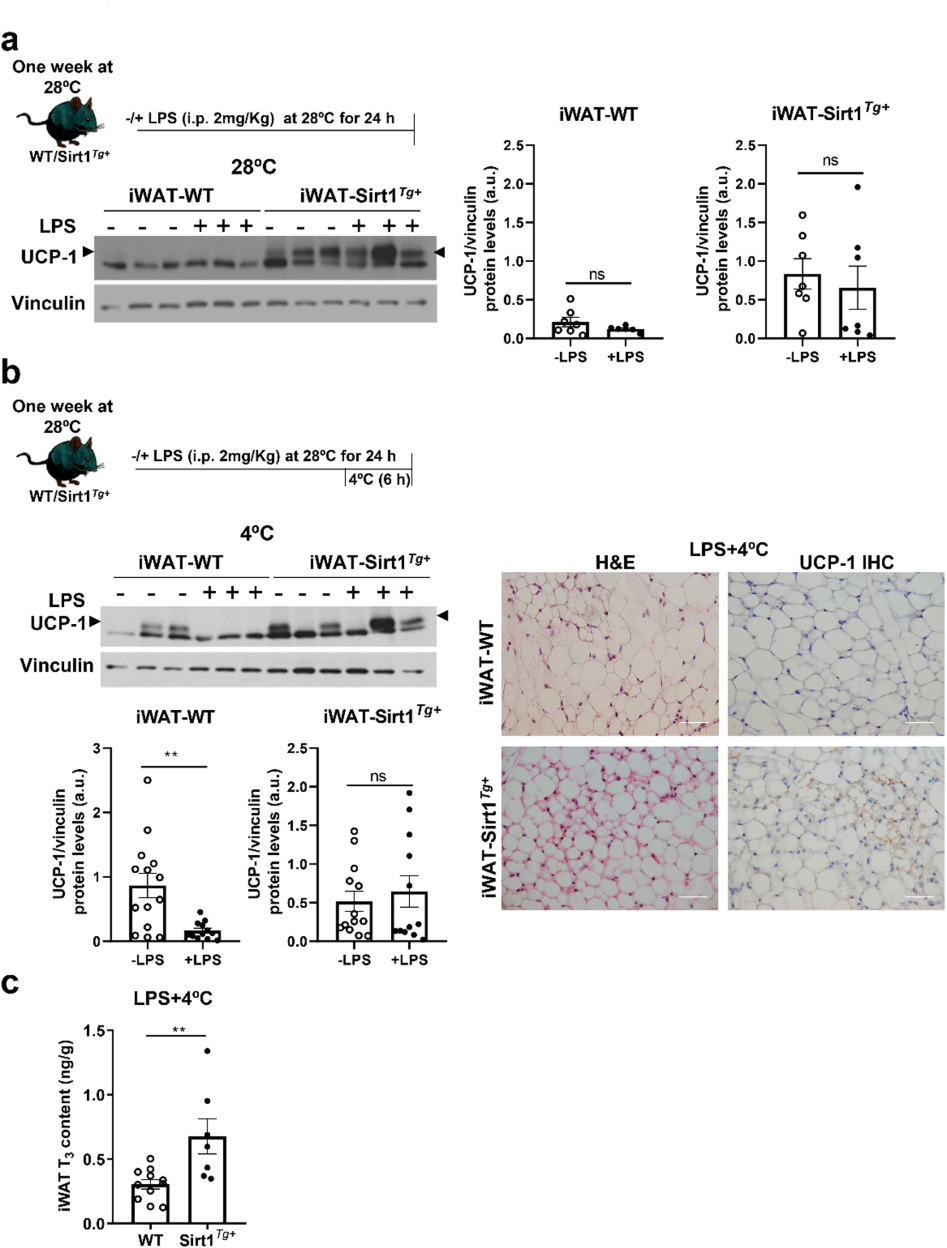
SIRT1 overexpression protects against the drop of UCP-1 levels in iWAT from LPS-injected mice. (**a**) Experimental protocol of LPS administration in mice. Representative UCP-1 Western blot and quantification in iWAT from WT and Sirt1^*Tg+*^ mice after 24 h of an i.p. LPS injection at thermoneutrality (*n* = 6–7 mice/group). (**b**) Experimental protocol of LPS administration and cold exposure in mice. (Left) representative Western blot and quantification of UCP-1 protein expression in iWAT from mice receiving or not an i.p. LPS injection for 24 h and exposed to 4 °C during the last 6 h (*n* = 12–14 mice/group). Arrowheads indicate the quantified band. Vinculin was used as loading control. (Right) Representative H&E staining and UCP-1 immunohistochemistry images in iWAT after LPS injection and cold exposure during the last 6 h. (**c**) T_3_ content in iWAT from WT or Sirt1^*Tg+*^ mice under these experimental conditions (*n* = 7–11 mice/group). Scale bars 50 μm. Data are expressed as mean ± SEM. Statistical analysis was conducted with the Mann–Whitney U test. **p < 0.01

Since inflammation negatively affects browning [11] we investigated the impact of LPS-mediated inflammation in iWAT from WT and Sirt1^*Tg+*^ mice (Fig. 2a).

At thermoneutral conditions, LPS did not modulate UCP-1 protein levels in either genotype. Of note, as also shown in Fig. 1b, higher UCP-1 protein levels were found in iWAT from Sirt1^*Tg+*^ mice and, interestingly, were maintained in LPS-injected mice. Then, we conducted these experiments in mice exposed to cold during the last 6 h of the LPS treatment (Fig. 2b). Under these conditions, a drop of UCP-1 protein levels was found in iWAT from WT mice while no differences in UCP-1 protein levels were found in Sirt1^*Tg+*^ mice. These results were corroborated by UCP-1 IHC and by visualizing features of iWAT activation in LPS-injected Sirt1^*Tg+*^ mice (Fig. 2b), pointing to a protection of SIRT1 overexpression against the deleterious effect of LPS upon cold exposure.

It has been reported that circulating thyroid hormone levels decrease in mice upon LPS administration [30]. In this context, our previous study provided evidence of the protection against the drop of T_3_ levels in BAT from Sirt1^*Tg+*^ mice exposed to LPS and cold. Based on that, we measured T_3_ levels in iWAT of mice exposed to the cold during the final 6 h of LPS treatment. As shown in Fig. 2c, mice overexpressing SIRT1 also had higher levels of T_3_ in iWAT (Fig. 2c), an effect which probably contributes to the maintenance of UCP-1 expression in proinflammatory conditions since it is well established that T_3_ positively regulates *Ucp1* mRNA in BAT [31]. Regarding iWAT, it has been demonstrated that T_3_ enhances thermogenesis by amplifying cAMP signaling [32].

### Sirt1 overexpression protects mice against the attenuation of insulin signaling by LPS in iWAT

**Fig. 3:**
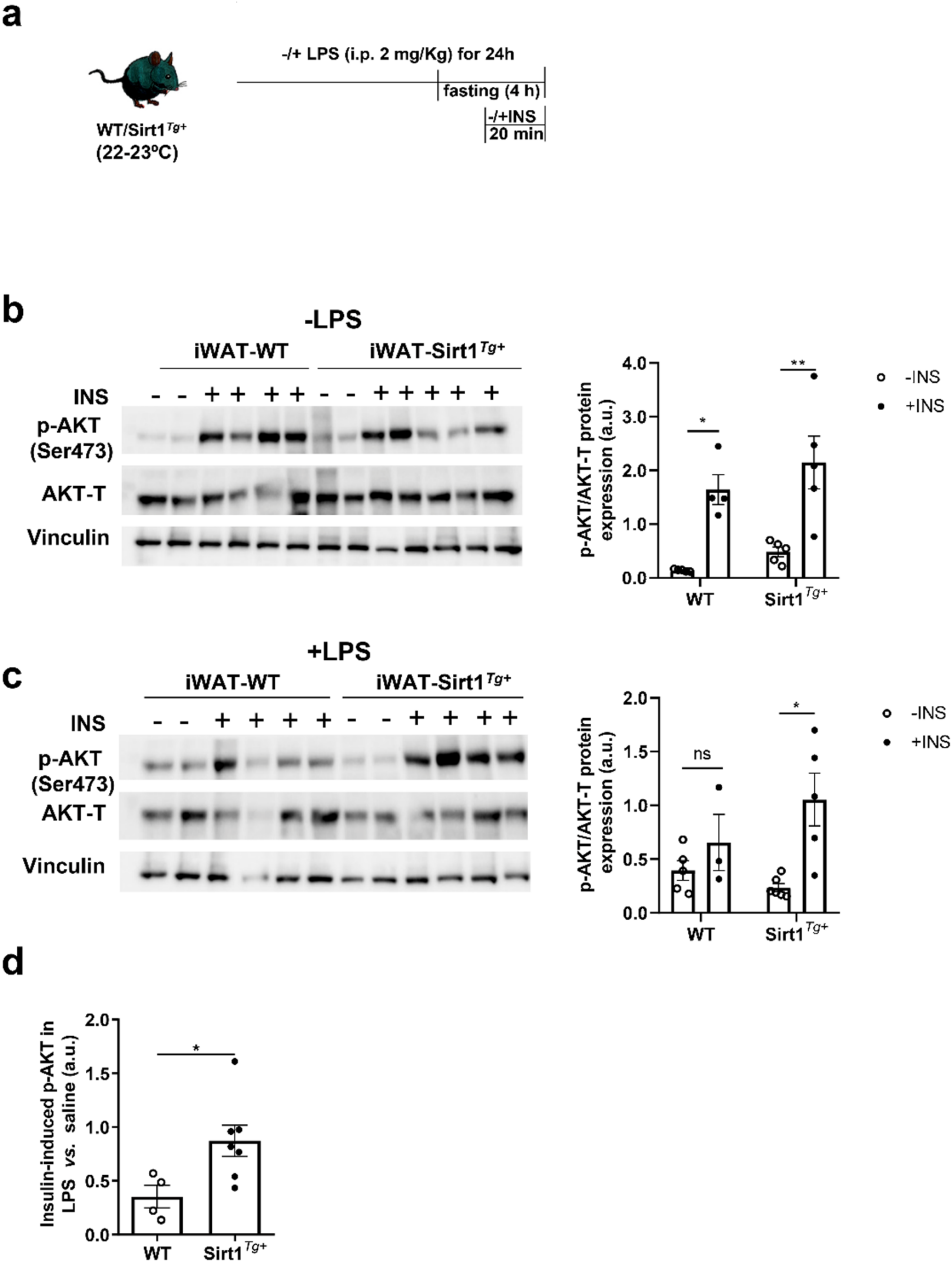
SIRT1 overexpression protects mice against the drop of insulin signaling in iWAT upon LPS injection. (**a**) Experimental protocol of LPS and insulin administration in mice. (**b**) Representative Western blots and quantification of AKT (Ser 473) phosphorylation levels in iWAT from WT and Sirt1^*Tg+*^ mice in basal conditions (-LPS) with or without 0.75 U/Kg of insulin (INS) stimulation (*n* = 4–5 mice/group). (**c**) Representative Western blots and quantification of AKT (Ser 473) phosphorylation after 2 mg/Kg of LPS (+ LPS) injection with or without INS stimulation (*n* = 3–6 mice/group). (**d**) Graph showing fold change of insulin- induced p-AKT in iWAT from mice injected with LPS vs. saline. Vinculin and total AKT (AKT-T) were used as loading controls. Data are expressed as mean ± SEM. Statistical analyses in **b** and **c** were performed by two-way ANOVA with Bonferroni´s post-hoc test. Statistical analysis in **d** was conducted with the Mann–Whitney U test. *p < 0.05, **p < 0.01

Subcutaneous adipocytes are insulin sensitive cells [7] and our previous work revealed the modulation of insulin signaling by SIRT1 in BAT under proinflammatory conditions [21]. Taking this into account we evaluated insulin signaling in iWAT from both genotypes of mice following the experimental protocol depicted in Fig. 3a.

WT and Sirt1^*Tg+*^ mice responded similarly to insulin in inducing AKT phosphorylation in this fat depot (Fig. 3b). Interestingly, upon LPS injection, insulin-induced AKT phosphorylation in iWAT from WT mice was impaired (Fig. 3c) whereas a marked insulin response was found in iWAT from Sirt1^*Tg+*^ mice (two-fold the response of the WT) (Fig. 3c and d), reinforcing the protective role of SIRT1 against iWAT inflammation.

### Differentiated iWAT adipocytes from mice overexpressing SIRT1 show higher thermogenic capacity in vitro

To analyze the cell-autonomous effect observed in iWAT from WT and Sirt1^*Tg+*^ mice we immortalized preadipocytes from this fat depot for both genotypes (iWA-WT and iWA-Sirt1^*Tg+*^, respectively). Moderate SIRT1 overexpression in iWA was assessed by qRT-PCR, being the relative expression 1.66 ± 0.044 in iWA- Sirt1^*Tg+*^ *versus* 1.02 ± 0.14 in iWA-WT (*p* < 0.05, *n* = 3). The iWAT preadipocyte cell lines were differentiated using 2 different media. Medium 1 was described by Armani et al. to differentiate primary inguinal white adipocytes [25] while medium 2, enriched in T_3_ and rosiglitazone, was used for beige cell differentiation as well [26]. The details of media composition are explained in Materials and Methods.

**Fig. 4:**
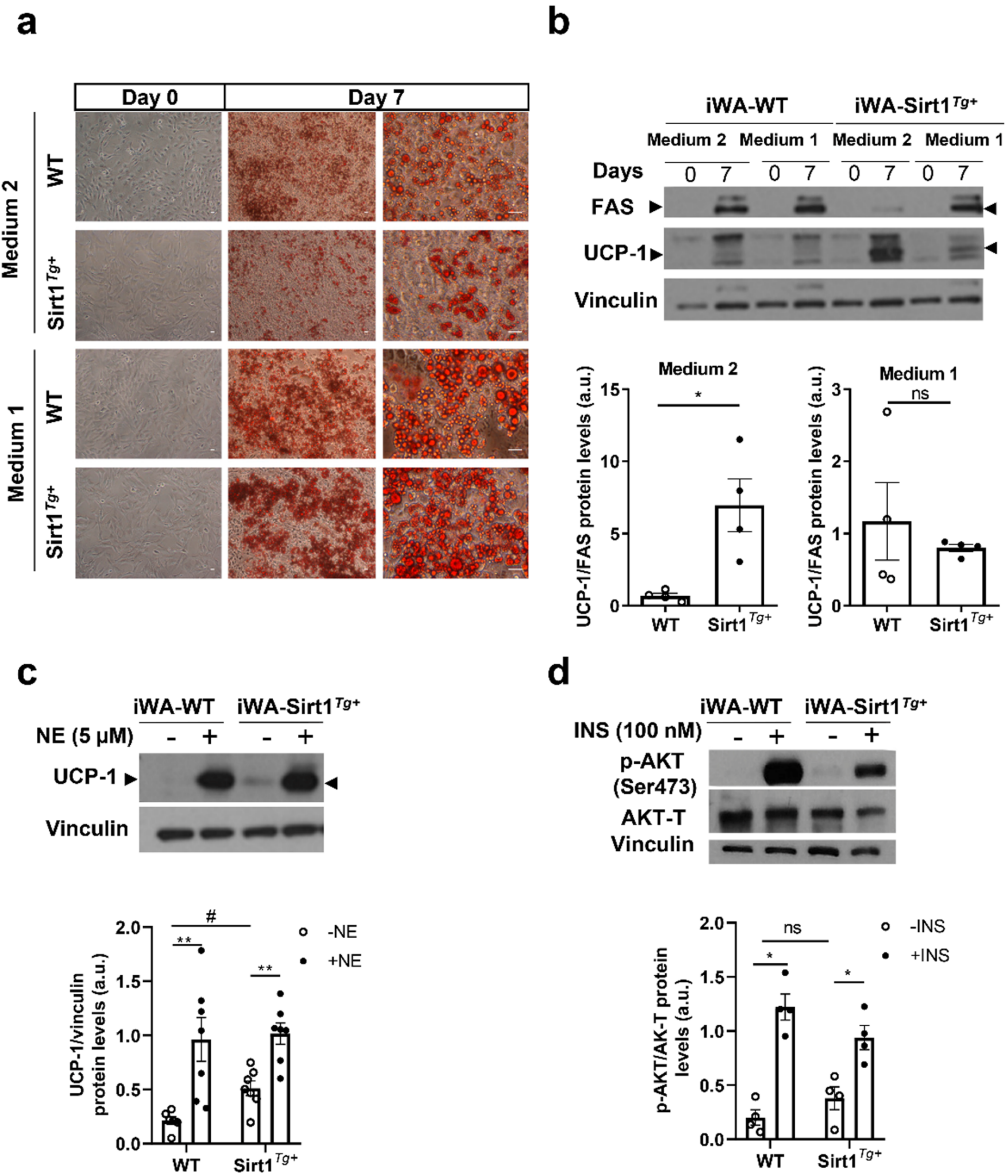
Differentiated iWA adipocytes from mice overexpressing SIRT1 show higher thermogenic capacity in vitro. (**a**) Representative microscopy images of iWA-WT and iWA- Sirt1^*Tg+*^ adipocytes before and after differentiation with medium 1 or medium 2 for 7 days and oil red O staining at day 7. Scale bars 200 μm. (**b**) Representative Western blots of UCP-1 and FAS and quantification of UCP-1/FAS ratio at day 7 in iWA-WT and iWA-Sirt1^*Tg+*^ adipocytes differentiated with medium 2 or 1 (*n* = 4 independent experiments). (**c**) Representative UCP-1 Western blot and quantification in iWA-WT and iWA- Sirt1^*Tg+*^ adipocytes differentiated with medium 2 stimulated or not with 5 µM NE during 18 h (*n* = 6–9 independent experiments). Arrowheads indicate the quantified band. (**d**) Representative Western blot and quantification of p-AKT (Ser 473) in iWA-WT and iWA-Sirt1^*Tg+*^ adipocytes differentiated with medium 2 and stimulated or not with 100 nM insulin for 15 min (*n* = 4 independent experiments). Vinculin and total AKT (AKT-T) were used as loading controls. Data are expressed as mean ± SEM. Statistical analysis was performed using the Mann–Whitney U test. *Comparisons between NE-treated and INS-treated and their untreated controls. ^#^Comparisons between same condition and different genotype. *,^#^*p* < 0.05,**p < 0.01

Both differentiation media allowed iWA preadipocyte differentiation as shown by oil red O staining of the lipid droplets (Fig. 4a); however, adipogenic differentiation was more evident in preadipocytes from both genotypes of cells differentiated with medium 1 that showed a higher number of lipid droplets (Fig. 4a).

Regarding the thermogenic differentiation pattern, the iWA-Sirt1^*Tg+*^ adipocytes differentiated with medium 2 showed higher UCP-1 protein levels in comparison to cells differentiated with medium 1 (Fig. 4b). Interestingly, UCP-1 levels in SIRT1 overexpressing iWA differentiated in medium 2 were higher despite of the lower number of cells with lipid droplets, as reflected at the molecular level by higher UCP-1/FAS ratio compared with WT adipocytes (Fig. 4b). These results correlate with the higher amount of UCP-1 found in iWAT from Sirt1^*Tg+*^ mice under basal conditions (Fig. 1b). Based on this, and on the higher capacity of the iWA-WT to respond to NE when differentiated in medium 2 (Supplemental Fig. 1), we performed further experiments in iWA-WT and iWA-Sirt1^*Tg+*^ differentiated with medium 2 to decipher the cell-autonomous effects of SIRT1 overexpression.

The capacity of iWA-WT and iWA-Sirt1^*Tg+*^ adipocytes to respond to two relevant physiological stimuli in adipose tissue, NE and insulin, was then tested. Figure 4c shows higher basal UCP-1 levels in iWA-Sirt1^*Tg+*^ adipocytes, but comparable UCP-1 levels after NE treatment (5 µM, 18 h) in both genotypes. Regarding insulin responsiveness, differentiated iWA-WT and iWA-Sirt1^*Tg+*^ adipocytes were stimulated with 100 nM insulin for 15 min. As shown in Fig. 4d, no differences were found in AKT phosphorylation in unstimulated cells. The response to insulin in inducing AKT phosphorylation in iWA-Sirt1^*Tg+*^ adipocytes was slightly (non-significant) lower compared to that of iWA-WT cells, an effect probably related to the lower adipogenic differentiation previously observed with oil red O staining (Fig. 4a).

### SIRT1 overexpression ameliorated the drop of NE and insulin responses under proinflammatory conditions in differentiated iWA

**Fig. 5:**
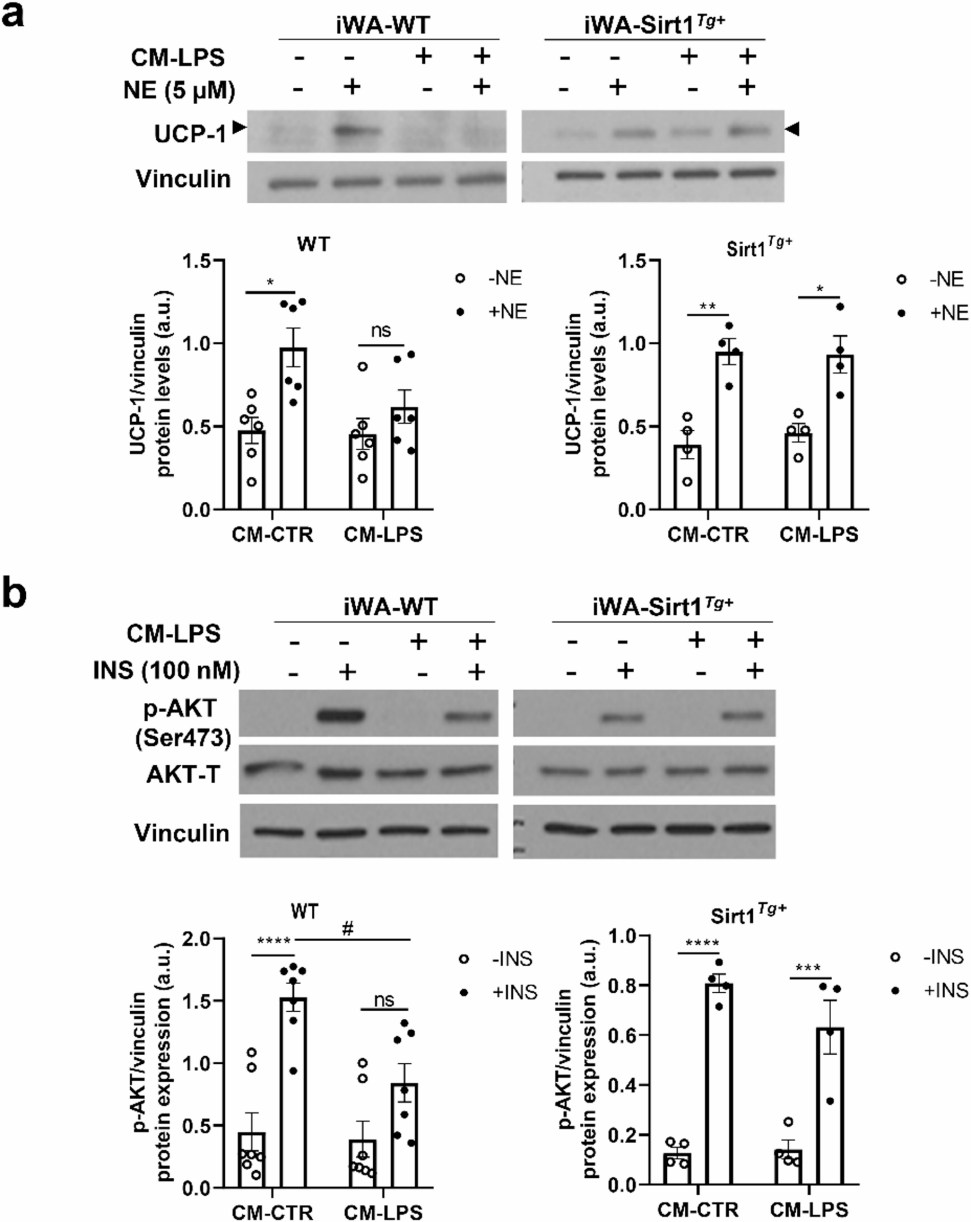
SIRT1 overexpression ameliorates the drop of NE and insulin responses under proinflammatory conditions in differentiated adipocytes from iWAT. (**a**) Representative Western blots and quantification of UCP-1 levels in iWA-WT and iWA-Sirt1^*Tg+*^ adipocytes in the presence of CM-CTR or CM-LPS and stimulated or not with 5 µM NE during 18 h (*n* = 4 independent experiments). Arrowheads indicate the quantified band. (**b**) Representative Western blot and quantification of p-AKT (ser473) levels in iWA-WT and iWA- Sirt1^*Tg+*^ adipocytes in the presence of CM-CTR or CM-LPS and stimulated or not with 100 nM insulin during 15 min (*n* = 4–7 independent experiments). Vinculin or total AKT (AKT-T) were used as loading controls. Data are expressed as mean ± SEM. Statistical analyses were performed by two-way ANOVA with Bonferroni´s post-test. *Comparisons between NE-treated and INS-treated and their untreated controls. ^#^Comparisons between same condition and different CM. *^,#^*p* < 0.05, **p < 0.01, ***p < 0.001, ****p < 0.0001

We next addressed whether inflammation could modulate NE and insulin responses in differentiated iWA-WT and iWA-Sirt1^*Tg+*^ adipocytes. For this goal, we generated a proinflammatory CM collected from Raw 264.7 macrophages stimulated or not with LPS (CM-LPS and CM-CTR, respectively). The CM were used to treat iWA-WT and iWA-Sirt1^*Tg+*^ for 18 h. To analyze the response to NE of the two genotypes of iWA, cells were co-treated with NE and CM-LPS or CM-CTR during 18 h. As shown in Fig. 5a, UCP-1 protein levels increased in iWA-WT exposed to CM-CTR in the presence of NE. However, this effect was suppressed in iWA-WT co-treated with CM-LPS and NE. By contrast, the response to NE in elevating UCP-1 levels was preserved in iWA-Sirt1^*Tg+*^ in the presence of CM-LPS in line with the in vivo results in iWAT from mice injected with LPS and exposed to the cold (Fig. 2b).

Similarly, a protection by SIRT1 overexpression was found in iWA stimulated with insulin during the last 15 min of the treatment with the CM-LPS or CM-CTR (Fig. 5b), also in correlation with the results in vivo (Fig. 3c and d).

### Proinflammatory conditions further increase SIRT1 protein levels in iWAT and adipocytes overexpressing SIRT1

**Fig. 6:**
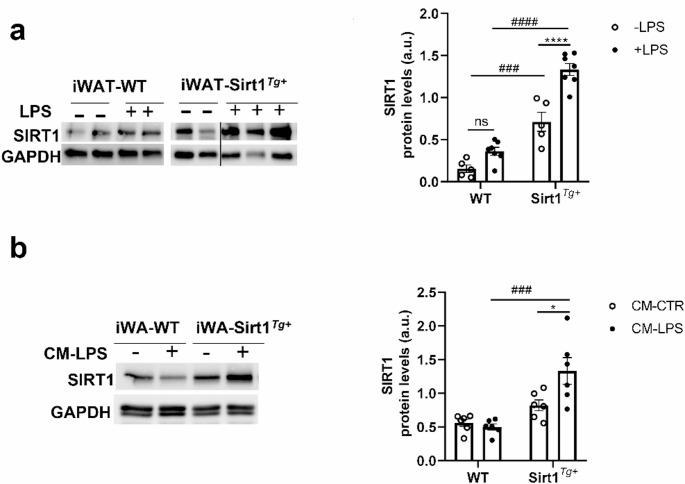
LPS increases SIRT1 protein levels in iWAT and iWA with SIRT1 overexpression. (**a**) Representative SIRT1 Western blots and quantification in iWAT from WT and Sirt1^*Tg+*^ mice with or without LPS injection (*n* = 5–7 mice/group). (**b**) Representative SIRT1 Western blot and quantification in iWA-WT and iWA-Sirt1^*Tg+*^ adipocytes differentiated with medium 2 in the presence of CM-CTR or CM-LPS (*n* = 6 independent experiments). GAPDH was used as loading control. Data are expressed as mean ± SEM. Statistical analyses were performed by two-way ANOVA with Bonferroni´s post-hoc test. *Comparisons between LPS-treated or CM-LPS-treated and LPS-untreated or CM-CTR, respectively. ^#^Comparisons between same condition and different genotype. *p < 0.05, ^###^*p* < 0.001, ^****,####^*p* < 0.0001

To further investigate the protective role of SIRT1 in iWAT under proinflammatory conditions we analyzed SIRT1 protein levels in iWAT samples collected from mice exposed to LPS for 24 h. As shown in Fig. 6a, in iWAT from WT mice, SIRT1 protein levels were modestly elevated upon 24 h of LPS treatment. Interestingly, Sirt1^*Tg+*^ mice showed a significant SIRT1 upregulation upon LPS injection (Fig. 6a). Likewise, iWA-Sirt1^*Tg+*^ adipocytes exposed to CM-LPS showed significant higher SIRT1 levels compared to those of iWA-WT cells (Fig. 6b).

## Discussion

Several studies have implicated SIRT1 in a variety of physiological and pathological processes, including cell cycle regulation, mitochondrial biogenesis, lipid and glucose metabolism, and energy homeostasis [16]. Importantly, losing weight in morbidly obese individuals resulted in a significant induction of SIRT1 in subcutaneous adipose tissue [33]. Similarly, other studies in preclinical models have demonstrated a role for adipose tissue SIRT1 in protecting against metabolic damage associated with obesity [34, 35]. Regarding iWAT, treatment of obese mice with the SIRT1 activator resveratrol upregulate SIRT1 levels in parallel with a reduction of several inflammatory markers [36], suggesting a therapeutic benefit of SIRT1 activation in this fat depot. On the other hand, it is widely known that browning of iWAT improves insulin resistance and increases energy expenditure as reviewed [37]. In this regard, overexpression of SIRT1 in mice fed a HFD increases browning and prevents diabetes [18, 35].

Endotoxemia is a key contributor to obesity-induced metainflammation [38, 39]. In fact, LPS levels in circulation are increased in *db/db* mice [38, 40]. We recently reported that moderate SIRT1 overexpression protects mice against LPS-induced pro-inflammatory signaling cascades, insulin resistance and reduced thermogenic responses upon cold exposure in BAT [21]. Additionally, in vitro experiments in brown adipocytes differentiated with a T_3_-enriched medium and then exposed to a macrophage-derived pro-inflammatory CM, revealed that only cells with SIRT1 overexpression maintained insulin and noradrenergic responses. These findings suggest that SIRT1 activators could be beneficial to combat metainflammation in this tissue. Based on these previous findings, herein we have provided evidence for the protective effect of moderate SIRT1 overexpression against the deleterious effects of LPS-mediated inflammation in iWAT, an issue not investigated so far.

Our results show higher UCP-1 levels in iWAT from Sirt1^*Tg+*^ mice housed in thermoneutrality, pointing to the ability of SIRT1 to upregulate UCP-1 levels in this fat depot even in the absence of thermal stress. Of relevance, thermoneutrality housed mice provide a more accurate model of the human metabolic phenotype than mice housed at RT as reported [41]. The increase in iWAT UCP-1 levels in Sirt1^*Tg+*^ mice under basal conditions is likely a cell autonomous effect as evidenced by the increased UCP-1 expression levels in differentiated inguinal white adipocytes overexpressing SIRT1 (iWA-Sirt1^*Tg+*^) with a protocol with higher thermogenic/lower adipogenic differentiation capacity as we previously reported in brown adipocytes [21]. Our results are consistent with the work of Zhou* et al.* [42] describing that SIRT1 overexpression limits adipogenesis in mesenchymal stem cells and the work of Kim* et al.* showing that the flavonoid Fisetin induces SIRT1 expression while inhibiting early adipogenesis in 3T3-L1 adipocytes [43]. Also, in hepatocytes SIRT1 activation significantly reduces lipid accumulation [44]. On the other hand, the effect of SIRT1 overexpression in the thermogenic pattern of iWA is likely related to its ability to deacetylate PPARγ, a transcription factor and master regulator of brown remodeling of iWAT, which is required to recruit the coactivator of the thermogenic program *Prdm16* [45]. Interestingly, the higher thermogenic differentiation pattern of iWA in medium 2 is also due, at least in part, to the enrichment with rosiglitazone, a PPARγ agonist that promotes *Prdm16* stabilization [46]. Also, the higher content of T_3_ in medium 2, which stabilizes *Ucp1* mRNA [47, 48], must also be taken into account.

We conducted experiments in mice exposed to cold (4ºC) for 6 h and, surprisingly, Sirt1^*Tg+*^ mice show comparable iWAT UCP-1 expression levels to WT mice suggesting that, at least in our mouse model with moderate SIRT1 overexpression, the maximal capacity of SIRT1 to upregulate UCP-1 is achieved at thermoneutrality. Another possible explanation for these results is the short duration of cold exposure (6 h), which differs from the overnight exposure reported in the study by Qiang et al. [18] in mice with loss of function of the SIRT1 inhibitor Deleted in Breast Cancer-1 (Dbc^−/−^ mice) and also in mice overexpressing SIRT1 (SIRBaco mice), in which higher UCP-1 induction was shown. Notably, similar UCP-1 levels were found in iWA-WT or iWA-Sirt1^*Tg+*^ after treatment with NE for 18 h in line with the in vivo results. Herein we also show that, under basal conditions, iWAT from Sirt1^*Tg+*^ mice responded to insulin stimulation as the WT control mice but, in this case, the in vitro response to insulin of iWA-Sirt1^*Tg+*^ adipocytes was slightly attenuated. This effect could be due to the presence of less adipocytes with an adipogenic differentiated phenotype, since it is well known that the expression levels of the insulin receptor, and consequently insulin signaling responses, gradually increase during adipogenic differentiation [49].

As mentioned above, WAT is the main tissue affected by the detrimental effects of obesity-associated meta-inflammation [50]. However, despite the intensive research in this area, it is still unclear whether adipose tissue dysfunction creates a low-grade chronic inflammatory state or whether inflammation leads to adipose tissue dysfunction. Our results in mice injected with LPS to mimic this inflammatory component showed that SIRT1 overexpression protects against the drop of both insulin and NE-mediated responses in iWAT, an effect clearly recapitulated in vitro in iWA adipocytes exposed to CM-LPS. These results are in line with our previous work [21] demonstrating the beneficial effects of SIRT1 overexpression in insulin/NE responses in vivo in BAT and in vitro in brown adipocytes differentiated with medium 2 and exposed to LPS or CM-LPS, respectively, due to an anti-inflammatory metabolic signature. It is noteworthy to highlight that intra-iWAT T_3_ levels were higher in Sirt1^*Tg+*^ mice exposed to LPS and cold, as occurred in BAT [21]. As mentioned above, T_3_ stabilizes *Ucp1* mRNA contributing to sustain UCP-1 levels upon SIRT1 overexpression under inflammatory and cold conditions. Importantly, herein we show that LPS increases SIRT1 expression in iWAT, particularly in mice with moderate SIRT1 overexpression and this effect is recapitulated in vitro in iWA- Sirt1^*Tg+*^ adipocytes exposed to CM-LPS. At present we cannot provide an explanation for such effect, but LPS-mediated SIRT1 upregulation has been described in mouse placental tissues and human trophoblasts [51]. In a pro-inflammatory context it has been reported that LPS induces SIRT1 in bone marrow-derived macrophages by IFN-β mediated JAK-STAT pathway [52]. By contrast, another study identified SIRT1 as a direct target of miR-181a-5p and it was up-regulated in LPS-treated Raw 264.7 macrophages, thereby resulting in a decrease in SIRT1 levels [53]. Interestingly, in our settings SIRT1 levels were increased in iWAT from Sirt1^*Tg+*^ mice that already showed a moderate SIRT1 overexpression. These results suggest first that LPS-mediated SIRT1 up-regulation might be a compensatory response to protect iWAT against inflammation and, second, that a threshold of SIRT1 levels is needed to preserve insulin and NE-mediated responses in iWAT and this is only achieved in Sirt1^*Tg+*^ mice. The fact that CM-LPS-mediated SIRT1 up-regulation is more evident in iWA-Sirt1^*Tg+*^ adipocytes point to adipocyte-specific mechanisms under this condition that might also contribute to the potential mechanisms associated to iWAT macrophages. This interesting hypothesis requires further investigation.

Overall, our work has shed light on the protective effect of SIRT1 overexpression against the deleterious effects of LPS, which is elevated in the circulation of obese individuals and decreases after bariatric surgery [54], in iWAT browning and insulin sensitivity. However, limitations such as the short-term exposure of mice to the pro-inflammatory stimulus of LPS and to the cold make necessary future studies in experimental models that more closely recapitulate human metainflammation. Furthermore, the analysis of visceral WAT is needed to determine the potential benefits of SIRT1 overexpression in this fat depot, which is highly sensitive to metainflammation [55]. Additionally, the impact of SIRT1 overexpression on the profile of recruited and/or resident immune cell populations in iWAT in mice subjected to LPS injection, either with or without cold exposure, deserves further research.

The potential translation of our preclinical results is supported by various human studies that have evaluated *SIRT1* gene expression in peripheral blood mononuclear cells (PBMCs) in different contexts. In healthy Korean individuals, the expression of *SIRT1* in PBMCs negatively correlates with visceral adiposity [56]. In a disease context, Crujeiras et al. found increased *SIRT1* expression levels in obese individuals following calorie restriction intervention [57]. Moreover, Liu et al. reported reduced *SIRT1* expression in the PBMCs of patients with type 2 diabetes and cognitive impairment [58]. Similarly, insulin resistance and subclinical atherosclerosis have been associated with low *SIRT1* gene and protein expression in PBMCs [59]. Another study reported an inverse correlation between SIRT1 expression in subcutaneous WAT from lean and obese individuals and monocyte/macrophage markers such as CD14, CD86 and CX3CL1 [20]. Taken together, these and other human studies strongly suggest that SIRT1 expression in circulating blood cells may represent a valuable marker for metabolic diseases.

In conclusion, our results have identified a role for SIRT1 as a potential therapeutic target against inflammation-mediated attenuation of insulin- and NE-related responses in iWAT.

## Electronic supplementary material

Below is the link to the electronic supplementary material.

Supplementary Material 1

## Data Availability

No datasets were generated or analysed during the current study.
