# Supplementary material for: Sirtuin 1 overexpression in mice preserves insulin and thermogenic responses in subcutaneous inguinal white adipose tissue under proinflammatory conditions
Source: J Physiol Biochem. 2025 Aug 4;81(4):1019–35. doi: 10.1007/s13105-025-01109-3 (PMC12738626; doi:10.1007/s13105-025-01109-3)
Supplement: Supplementary file 1 — Supplementary Material 1 [file 13105_2025_1109_MOESM1_ESM.docx]

**Sirtuin 1 overexpression in mice preserves insulin and thermogenic responses in subcutaneous inguinal white adipose tissue under proinflammatory conditions**

Patricia Vázquez ^1,2,3*^, Carmen Escalona Garrido^1,2#^, Nuria Pescador^1,2#^, Ana B. Hitos^1,2^, Daniel González-Moreno^1^, Ángela de Benito-Bueno^1^, Elena Sierra-Filardi^4^, Patricia Boya^4^, Ana Montero-Pedrazuela^1^, Ana Guadaño-Ferraz^1^, Ángela M. Valverde^1,2*^

^1^Instituto de Investigaciones Biomédicas “Sols-Morreale” (IIBm, CSIC-UAM), Madrid, Spain.

^2^CIBER de Diabetes y Enfermedades Metabólicas (CIBERDEM), ISCIII, Madrid, Spain.

^3^Departamento de Bioquímica y Biología Molecular. Facultad de Medicina. Universidad Complutense de Madrid.

^4^Departamento de Biología Celular y Molecular, Centro de Investigaciones Biológicas Margarita Salas (CIB, CSIC), Madrid, Spain.

^#^equal contributors

D. G-M current address: Research Institute of the Hospital Universitario 12 de Octubre (imas12), Madrid, Spain.

P.B. current address: Department of Neuroscience and Movement Sciences, University of Fribourg, Switzerland.

*Corresponding authors

Email Address of the Corresponding Authors:

pvazquezperez@ucm.es; avalverde@iib.uam.es


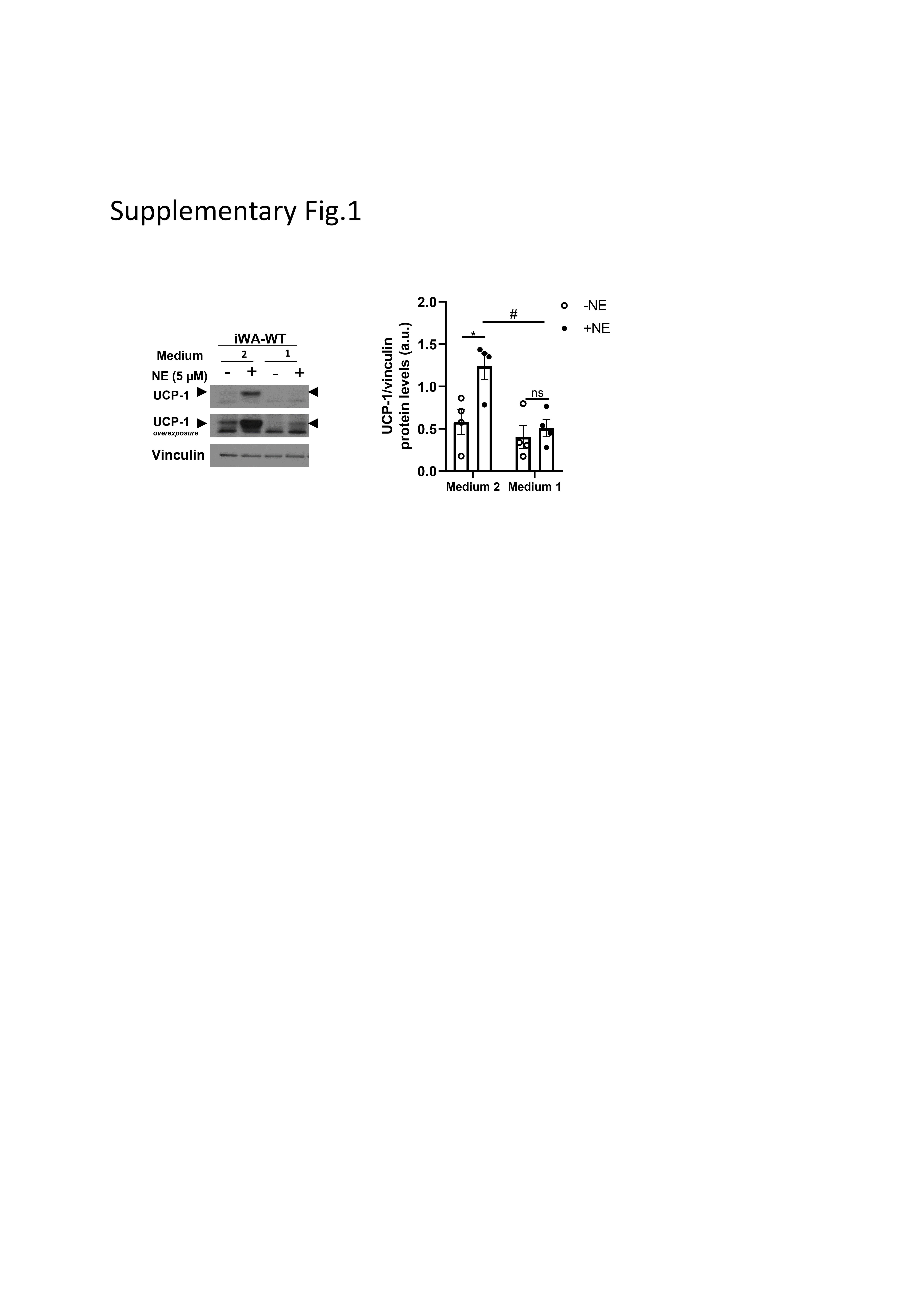


**Supplementary Fig. 1 *Differentiated iWA-WT adipocytes with medium 2 show higher response to NE in inducing UCP-1*** a) Representative Western blot of UCP-1 and quantification in iWA-WT adipocytes differentiated with medium 1 and 2 stimulated or not with 5 µM norepinephrine (NE) during 18 h (n=4 independent experiments). Arrowheads indicate the quantified UCP-1 band. Vinculin was used as loading control. Data are expressed as mean ± SEM. Statistical analysis was performed using two-way ANOVA. *Comparisons between NE-treated and the untreated controls. ^#^Comparisons between same condition and different media. *,^#^p<0.05.
